# Comparison of indocyanine green and methylene blue use for axillary reverse mapping during axillary lymph node dissection

**DOI:** 10.1002/mco2.31

**Published:** 2020-09-17

**Authors:** Jun‐Dong Wu, Zun Wang, Huan‐Cheng Zeng, Li‐Fang He, Yong‐Qu Zhang, Guang‐Sheng Huang, Fan Zhang, Xiao‐Long Wei, Wen‐He Huang, Guo‐Jun Zhang

**Affiliations:** ^1^ The Breast Center Cancer Hospital of Shantou University Medical College Shantou Guangdong China; ^2^ Guangdong Provincial Key Laboratory for Breast Cancer Diagnosis and Treatment Cancer Hospital of Shantou University Medical College Shantou China; ^3^ ChangJiang Scholar's Laboratory Shantou University Medical College (SUMC) Shantou China; ^4^ The Central Laboratory Cancer Hospital of Shantou University Medical College Shantou China; ^5^ Department of Pathology Cancer Hospital of Shantou University Medical College Shantou China; ^6^ Cancer Center & Department of Breast and Thyroid Surgery Xiang'an Hospital School of Medicine Xiamen University Xiamen China

**Keywords:** axillary lymph node dissection, axillary reverse mapping, breast cancer, fluorescence imaging, indocyanine green, lymphedema

## Abstract

Axillary reverse mapping (ARM) is a technique to identify arm lymphatic drainage during axillary lymph node dissection (ALND). This study compared the feasibility of ARM using indocyanine green (ICG) or methylene blue (MB), and accessed the oncologic safety of the procedure. Overall, 158 patients qualified for ALND were enrolled. The characteristics of ARM‐identified nodes were recorded with ICG (n = 78) or MB (n = 80) visualization. Fine‐needle aspiration cytology (FNAC) of the nodes were performed and validated by histologic analysis. The nodal identification rate in the ICG group significantly surpassed that of the MB group (87.2% vs 52.5%, *P* < .05) with fewer complications. Note that 10.9% of the patients had metastatic involvement of the ARM‐identified nodes. Also 80% of the positive nodes were found in areas B and D, while the ARM‐identified nodes mainly located in area A. All the 51 nodes diagnosed as negative of malignancy by FNAC were free of metastasis. Nodal metastasis was significantly correlated with extensive nodel involvement, advanced disease, and the characteristics of identified nodes. In conclusion, ICG appears superior to MB for ARM nodes identification. FNAC, together with the features of primary tumors and ARM nodes, can delineate which nodes could be preserved during ALND.

## Graphical abstract

Axillary reverse mapping (ARM) using indocyanine green is feasible with a higher identification rate of ARM nodes and fewer complications. Note that 10.9% of the patients had metastatic involvement of the ARM‐identified nodes. FNAC, together with the features of primary tumors and ARM nodes, could delineate the patient profiles who can use ARM safely.

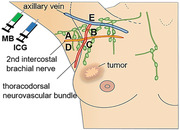

## 1 INTRODUCTION

Approximately 30% of patients with breast cancer harbor regional lymph node metastases.^1^, ^2^ Although complete level I and II axillary lymph node dissection (ALND) remains the standard of care in this setting, the associated morbidity is significant due to upper limb effects, including lymphedema, seroma, dysfunction, and sensory loss. Lymphedema of the upper extremity is particularly debilitating and ostensibly results from disrupted lymphatic drainage at the axilla. In a systematic review and meta‐analysis, the overall incidence of arm lymphedema obtained from pooled patients was 13.6‐20.2%.^3^ Axillary reverse mapping (ARM) has been used to trace lymphatics and lymph nodes from the upper extremity,^4^, ^5^ so that drainage is preserved and resultant lymphedema is curtailed.

Blue dye is the most common tracer used for mapping the upper extremity lymphatic drainage at the axilla.^4^, ^6^, ^7^ It was first coupled with ARM in 2007,^4^, ^8^ and many studies have since described its implementation for ARM during ALND.^9^, ^10^, ^11^, ^12^, ^13^, ^14^, ^15^ However, the use of blue dye alone for ARM has proved inconsistent, yielding nodal identification rates of 39‐90%. This wide range is perhaps attributable to the variable experience of surgeons with the ARM technique or the blue dye itself.^7^ At the same time, it is hard to visualize the lymphatic networks of ARM.^4^, ^8^

Indocyanine green (ICG) is used as the florescent contrast agent for the visualization of lymph nodes and lymphatics. It has been used to perfuse tissue, administer targeted therapies, and define anatomic elements (i.e., neurovascular and ophthalmic) intraoperatively, particularly during routine sentinel lymph node detection.^16^ So far, only several studies have reported the use of ICG fluorescence imaging for ARM, showing high and stable visualization rates for both nodes and lymphatics during ALND.^17^, ^18^, ^19^ However, there is a paucity of study to compare the nodal identification rates by ICG or methylene blue (MB) during ARM procedure.

ARM is based on the premise that the upper limb and breast lymphatics, which drain into the axilla, may be differentiated during surgery to mitigate lymphedema of the arm through lymphatic sparing. However, reports indicate that in a proportion of patients, nodes differentiated by ARM have shown metastases.^20^, ^21^ The oncologic safety of ARM is therefore in question. Nevertheless, intraoperative assessment to exclude metastasis in lymph nodes identified by ARM should instill confidence to ensure their preservation.

In the present study, ICG or MB was used to identify lymph nodes by ARM and to correlate features of tumor‐bearing nodes with the clinicopathologic characteristics of primary lesions. We also performed fine needle aspiration cytology (FNAC) of ARM‐identified nodes, comparing outcomes with final postoperative histopathology findings.

## 2 MATERIALS AND METHODS

### 2.1 Patient population

A prospective randomized study was conducted, with the recruitment of 158 women scheduled for ALND between February 2015 and June 2018 at the Breast Center, Cancer Hospital of Shantou University Medical College. The inclusion criteria conformed to the 7th Edition of the American Joint Committee on Cancer (AJCC) Cancer Staging Manual^22^ as follows: (1) clinical TNM stage T1‐4 or N0‐3 breast cancer, (2) clinical or cytological (via FNAC) evidence of axillary node positivity or clinically negative axillary nodes with positive sentinel node biopsy, and (3) ALND after neoadjuvant chemotherapy (NAC) for locally advanced breast cancer. General information and clinical data collected from patients included age, body mass index (BMI), treatments, and molecular biomarkers.

All patients were randomly assigned to ARM using ICG (n = 78) or MB (n = 80). This study was approved by the ethics committee of the Cancer Hospital of Shantou University Medical College, adhering to the 1964 Declaration of Helsinki and all subsequent revisions. Each subject was informed of the study aims/potential risks and granted signed consent in advance of participation. The trial was registered at www.chictr.org.cn (identifier: ChiCTR2000033797).

### 2.2 ALND procedure

ALND was conducted following a standard protocol, removing all lymph node‐bearing tissue from the thoracodorsal neurovascular bundle laterally to the chest wall medially, from the axillary vein superiorly to the insertion of thoracodorsal vessels into the latissimus dorsi muscle distally, and from the anterior aspect of the axillary vein to the subscapularis muscle posteriorly. At the superior‐most aspect of the axillary dissection, just inferior to the axillary vein, the lymph node tissue immediately lateral to the thoracodorsal vein was also routinely taken. Level II lymph nodes posterior to the pectoralis minor muscle were included as part of the axillary specimen. Level III lymph nodes were removed if indicated for palpable disease.

### 2.3 ARM technique

Once general anesthesia was induced (∼20 min prior to ALND), 1 mL of 0.5% ICG (25 mg vials; Yichuang Pharmaceutical LLC, Dandong, China) was injected subcutaneously at the upper and inner aspects of the ipsilateral arm, or 2 mL of 1% MB (Jichuan Pharmaceutical LLC, Jiangsu, China) was injected into the medial intermuscular groove of the ipsilateral upper arm (Figure 3). The limb was then elevated and the injection site massaged (∼5 min). During ALND, fluorescence images using ICG were obtained by an invisible near‐infrared fluorescence imaging system (Photo Dynamic Eye; Hamamatsu Photonics Company, Hamamatsu, Japan) for identifying the ARM nodes and/or lymphatics. There are two key sections in the imaging system, the light source and the detector. The light source is a light emitting diode to emit light at the wavelength of 760 nm, which could excite ICG to emit a highly penetrative infrared fluorescence, and the detector is a charge‐coupled device camera with a filter used to filter out light with a wavelength below 820 nm and acquire the specific fluorescence of ICG. On the other hand, blue lymphatics/nodes were visible to the naked eye. All visualized lymphatic channels and nodes were recorded.

### 2.4 ARM nodal locations

**FIGURE 1:**
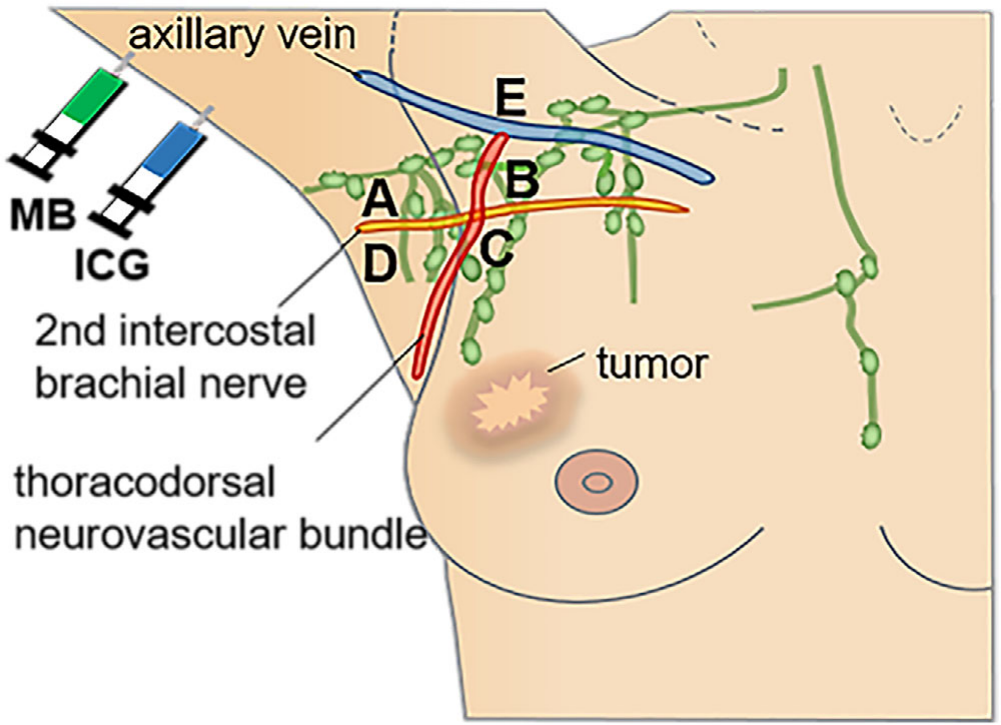
The location of nodes identified by axillary reverse mapping during axillary lymph node dissection and dye injection sites. According to axillary surgical landmarks (the axillary vein, thoracodorsal neurovascular bundle, and second intercostal brachial nerve), the axillary region was divided into five areas (A, B, C, D, and E)

Locations of all lymph nodes identified by ARM were delineated (Figure 1) with respect to axillary surgical landmarks, such as the axillary vein, thoracodorsal neurovascular bundle, and second intercostal brachial nerve. The five defined areas of ALND are as follows: (a) the area between the axillary vein and second intercostal brachial nerve, nearing the anterior edge of latissimus dorsi muscle; (b) the area adjacent and medial to area A and near the anterior serratus muscle; (c) the area below the second intercostal brachial nerve and near the anterior serratus muscle; (d) the area below the second intercostal brachial nerve, nearing the anterior edge of the latissimus dorsi muscle; and (e) the area above the axillary vein.

### 2.5 Pathologic evaluations

In addition to recording the numbers, shapes, sizes, and textures of nodes identified by ARM, intraoperative FNAC was regularly performed (using a 23‐gauge needle and a 10‐mL aspirating syringe) for immediate screening. The specimens were interpreted by two pathologists with proven expertise in breast cytology. Outcomes of FNAC were reported as negative, suspicious, or positive for malignancy, with some inadequate for diagnosis. When calculating estimates of accuracy, these lymph nodes diagnosed as suspicious was included as positive. Nodes identified by ARM were removed for routine histological processing and staining (hematoxylin and eosin).

### 2.6 Postoperative evaluation and follow‐up

Complications related to the ARM procedure, including skin tattoos, pain at injection sites, local skin reactions, and induration, were evaluated in all patients 1, 2, 4, 6, and 8 weeks after surgery and every 3–6 months thereafter.

### 2.7 Statistical analysis

Standard software (SPSS v16.0; SPSS Inc, Chicago, IL) was applied for all computations. Group differences were determined by independent *t*‐tests, using Pearson's *χ* ^2^ test or by Fisher's exact test to assess relations between ARM‐defined nodes and clinicopathologic variables. In two‐tailed testing, significance was set at *P* < .05.

## 3 RESULTS

### 3.1 Clinicopathologic characteristics of patients

**FIGURE 2:**
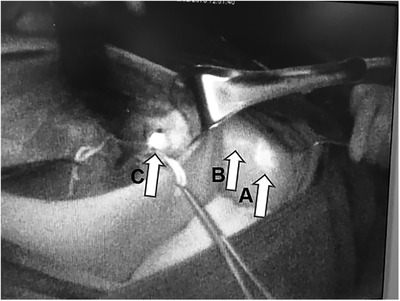
Axillary reverse mapping (ARM) procedure using indocyanine green (ICG): Arrow A, ICG injection site; Arrow B, the subcutaneous lymphatic ducts through the skin; Arrow C, axillary lymph nodes received the drainage of the arm lymphatics

**FIGURE 3:**
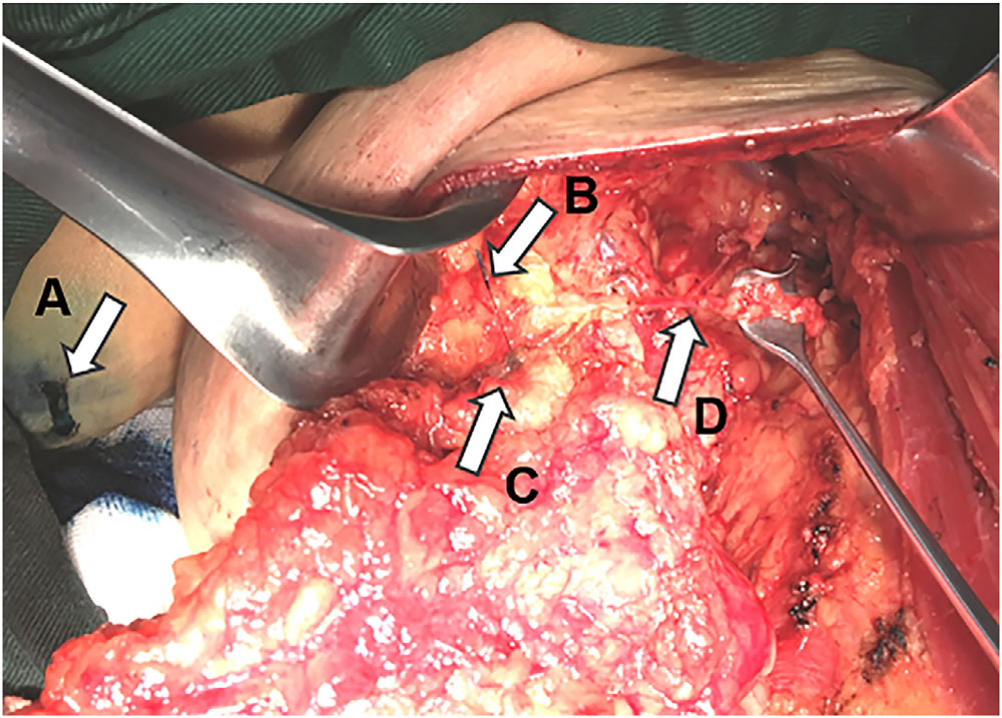
Axillary reverse mapping (ARM) procedure using methylene blue (MB): Arrow A, MB injection site; Arrow B, ARM of lymphatic ducts; Arrow C, ARM of lymph nodes; Arrow D: second intercostal brachial nerve

A total of 158 patients who underwent ALND between February 2015 and June 2018 for histologically diagnosed breast cancer were enrolled in this study. Among them, 78 (mean age, 54.76 ± 10.94 years) were injected with ICG for ARM (Figure 2), 16 of whom had received prior NAC, and another 80 patients (mean age, 53.01 ± 8.78 years) were injected with MB (Figure 3), 18 of whom had received prior NAC.

The clinicopathologic characteristics of the patients in the two groups are presented in Table S1. There were no significant group differences in terms of age, BMI, tumor size, pathologic grade, clinical lymph node status, hormonal receptor status, HER‐2 positivity, Ki‐67 index, molecular subtype, NAC, or clinical disease stage.

### 3.2 Significantly higher rate of ARM nodal identification in ICG (vs MB) group

In the ICG group, lymph nodes were successfully identified by ARM in 68 patients (87.2%) during ALND, compared with 42 (52.5%; *P *< .05) in the MB group. Furthermore, 108 nodes (mean, 1.59 ± 0.89) were obtained from the ICG recipients, compared with 61 (mean, 1.45 ± 0.80) from the patients administered MB. In the ICG group, the area distributions of ARM‐defined nodes in the patients were as follows: (a) 59.3% (64/108); (a) 25.9% (28/103); (c) 5.6% (6/108); (d) 8.3% (9/108); and (e) 0.9% (1/108). The corresponding distributions in the patients of the MB group were as follows: (a) 47.6% (29/61); (b) 29.5% (18/61); (c) 9.8% (6/61); (d) 13.1% (8/61); and (e) 0.0% (0/61; Table 1). Lymph nodes identified by ARM were thus largely confined to area A.

**TABLE 1:** Locations of ARM‐identified lymph nodes (mean ± SD)

	ICG group	MB group	*P* value
	(n = 108)	(n = 61)	
Range	1‐4	1‐4	
Mean ± SD	1.59 ± 0.89	1.45 ± 0.80	.428
Location*			
A	64 (59.3)	29 (47.6)	
B	28 (25.9)	18 (29.5)	
C	6 (5.6)	6 (9.8)	
D	9 (8.3)	8 (13.1)	
E	1 (0.9)	0 (0.0)	

Abbreviations: ARM, axillary reverse mapping; IG, indocyanine green; MB, methylene blue.

* Data expressed as numerical value (%).

In our study, 16 patients from the ICG group and 18 patients from the MB group received NAC. However, receiving NAC did not significantly affect the nodal identification rate by ARM in both the ICG and MB groups (Table S2).

We also accessed the effect of BMI on the detection rate of ARM lymph nodes, and found the difference was not statistically significant between three BMI subgroups in the ICG group or the MB group (Table S3).

### 3.3 Clinicopathologic factors associated with ARM‐identified nodal metastasis

Overall, nodal metastasis was detectable by ARM in 12 of 110 patients (10.9%), showing significant associations with larger tumors (*P* = .007), extensive nodal involvement (*P* < .001), and advanced TNM stage (*P* < .001; Table 2). There was no correlation between node metastases and other tumor parameters (i.e., histotype, grade, hormonal receptor status, molecular subtype, Ki‐67 index) or receiving NAC (Table 2). In addition, 80% of the positive nodes were primarily encountered in areas B and D (*P* < .001), correlating significantly with a rounded shape (*P* < .001), firmness (*P* < .001), and a diameter of > 1 cm (*P* = .003; Table 3).

**TABLE 2:** Association of clinicopathological factors with ARM‐identified nodal metastasis

	Node‐positive patients (n = 12)	Node‐negative patients (n = 98)	*P* value
NAC			.27
Yes	4 (33.3)	19 (19.4)	
No	8 (66.7)	79 (80.6)	
Pathologic T classification			.007**
pT1	0 (0.0)	21 (21.4)	
pT2	7 (58.3)	69 (70.4)	
pT3	3 (25.0)	4 (4.1)	
pT4	2 (16.7)	4 (4.1)	
Pathologic node classification			<.001***
pN1	3 (25.0)	81 (82.6)	
pN2	2 (16.7)	14 (14.3)	
pN3	7 (58.3)	3 (3.1)	
Histologic grade			.54
I	0 (0.0)	6 (6.1)	
II	7 (58.3)	35 (35.7)	
III	5 (41.7)	52 (53.1)	
Unknown	0 (0.0)	5 (5.1)	
Histotype			1.00
Ductal	11 (91.7)	87 (88.8)	
Lobular	0 (0.0)	3 (3.1)	
Other	1 (8.3)	8 (8.1)	
ER			.75
Negative	5 (41.7)	33 (33.7)	
Positive	7 (58.3)	65 (66.3)	
PRe			.77
Negative	6 (50.0)	44 (44.9)	
Positive	6 (50.0)	54 (55.1)	
HER‐2			.52
Negative	7 (58.3)	68 (69.4)	
Positive	5 (41.7)	30 (30.6)	
Ki‐67			.67
≤14	2 (16.7)	13 (13.3)	
>14	10 (83.3)	85 (86.7)	
Molecular subtype			.89
Luminal A	0 (0.0)	10 (10.2)	
Luminal B	8 (66.6)	56 (57.1)	
HER‐2+/ER‐/PR‐	2 (16.7)	17 (17.4)	
Triple negative	2 (16.7)	15 (15.3)	
Clinical stage			< .001***
I	0 (0.0)	10 (10.2)	
II	2 (16.7)	64 (65.3)	
III	10 (83.3)	24 (24.5)	

Data expressed as numerical value (%).

Abbreviations: ARM, axillary reverse mapping; ER, estrogen receptor; HER, human epidermal growth factor receptor; NAC, neoadjuvant chemotherapy; PR, progesterone receptor.

*P < .0; **P < .01; ***P < .001.

**TABLE 3:** Correlation of metastasis in ARM‐identified nodes

	Positive	Negative	*P* value
	(n = 18)	(n = 151)	
Area			
A	2 (11.1)	91 (60.2)	<.001***
B	12 (66.6)	34 (22.5)	
C	1 (5.6)	11 (7.3)	
D	3 (16.7)	14 (9.3)	
E	0 (0.0)	1 (0.7)	
Nodal shape			<.001***
Oval	4 (22.2)	70 (46.4)	
Round	14 (77.8)	43 (28.5)	
Not evaluated	0 (0.0)	38 (25.1)	
Texture of node			<.001***
Firm	11 (61.1)	12 (7.9)	
Hard	6 (33.3)	30 (19.9)	
Soft	1 (5.6)	69 (45.7)	
Not evaluated	0 (0.0)	40 (26.5)	
Diameter of node, mm			.003**
>15	6 (33.3)	18 (11.9)	
10–15	10 (55.6)	52 (34.4)	
<10	2 (11.1)	54 (35.8)	
Not evaluated	0 (0.0)	27 (17.9)	

Data expressed as numerical value (%).

Abbreviation: ARM, axillary reverse mapping.

*P < .05; **P < .01; ***P < .001.

### 3.4 Role of intraoperative FNAC in ARM evaluations of nodal metastasis

Intraoperative FNAC was performed to assess nodes detected by ARM for metastases (Figure S1). FNAC was assessed in a total of 44 ARM‐identified nodes in the ICG group (negative, 27; positive, 6; suspicious, 5; inadequate, 6) and a total of 40 in the MB group (negative, 24; positive, 6; suspicious, 6; inadequate, 4). For the 63 ARM nodes that could be assessed by FNAC, no discordance was noted between the cytological assessments by FNAC and the histological results. On the other hand, among the 11 nodes diagnosed as suspicious by FNAC, three nodes involved breast cancer metastasis while eight nodes were free of cancer cells (Table S4). In this setting, the specificity and sensitivity of FNAC was 86.4% (51/59) and 100% (15/15).

### 3.5 Fewer complications in ICG (vs MB) group

Postoperative complications from ARM were significantly fewer in the patients of the ICG (vs MB) group and were as follows: local skin reactions, 28.2% versus 66.3% (*P* = .004); pain at injection sites, 21.8% versus 71.3% (*P* < .001); and induration, 3.8% versus 57.5% (*P* < .001; Table S5). There was no obvious difference between the groups in terms of skin tattoos. All local skin reactions and pain dissipated within 2 weeks, whereas induration and skin tattoos of most patients disappeared within 1 year. Neither group experienced systemic allergic reactions.

## 4 DISCUSSION

To date, researchers have raised three main issues pertaining to ARM, questioning its adequacy with regard to lymphatic channel and nodal identification, inherent oncologic safety (especially in patients with advanced breast cancer), and utility in preventing lymphedema. In this prospective study, we have demonstrated that the use of ICG (rather than MB) for ARM yields satisfactory identification rates of nodes and/or lymphatics in node‐positive patients with breast cancer. NAC and BMI had no significant impact on ARM identification of nodes. We also determined that nodal location by ARM, tumor stage, and extent of lymph node involvement were closely correlated to the risk of metastasis in ARM‐identified nodes, which could be corroborated by intraoperative FNAC.

ICG fluorescence imaging has been used in other procedures for the mapping of the lymphatic system drainage,^23^, ^24^, ^25^ and we found it to be more sensitive than MB (87.8% vs 52.5%) during ARM, largely due to its optical properties. Upon excitation, ICG emits a highly penetrative infrared fluorescence (peak, ∼820 nm), with a higher signal‐to‐noise ratio than visible light, facilitating lymphatic visualization in deeper tissue and providing clearer definition from surrounding elements. ICG is therefore a promising replacement of MB. However, the dual usage of ICG and MB makes it possible to map the lymphatic system of the breast and upper arm simultaneously.^24^, ^25^

The impact of NAC on the rates of ARM‐identified nodes has remained controversial. Some studies seem to indicate that NAC has no effect on ARM detectability, although it is able to reduce the rates of metastasis in ARM‐identified nodes.^9^, ^12^ It has also been reported that NAC may lead to sclerosis of lymphatic channels, resulting in low detection rates.^26^ Although the nodal identification and metastasis rates that we determined via ARM were unchanged by NAC, our sample size was small and may be biased. A larger number of patients are needed to ascertain the consequences of NAC in this setting.

Different from the impact on the detection rates of sentinel lymph node,^27^ it is unknown that whether BMI as a significant variant affects the successful identification of ARM nodes. We evaluated the BMI's impact on the detection of ARM nodes, and did not find significant association of BMI with the identification in both the ICG group and MB group. In previous studies, Ponzone and colleagues found that BMI seemed to be negatively correlated with ARM identification, but there was no statistical difference.^20^ In contrast, Pavlista and colleagues found that lymphatics of the upper extremity were more difficult to visualize in overweight patients in an anatomic study of the lymphatic drainage of the upper extremity.^28^ Therefore, it is necessary to further explore whether BMI is associated inversely with the identification of ARM nodes.

Oncologic safety is another concern of ARM. In the present study, cancer metastases of ARM‐identified nodes were found in 10.9% (12/110) from clinically node‐positive breast cancer patients. Previous reports have cited demonstrable involvement of ARM‐identified nodes at rates of 8.7‐43% in patients with clinical node positivity.^20^, ^21^, ^29^ In a relatively large study, ARM‐identified nodes were involved in 12 (10%) of 120 patients who underwent ARM during ALND,^12^ which is similar with our study. We also found that ARM‐identified nodal metastases occupied areas B and D preferentially, consistent to the results of previous studies.^18^, ^30^, ^31^, ^32^ It could be explained that, in area B, the relation between metastasis and location signifies a crossover between ARM‐identified nodes and lymphatic drainage nodes of the breast, especially sentinel lymph nodes,^21^ whereas ARM‐identified nodal metastasis in area D is an apparent harbinger of cancer progression.^18^ From a safety perspective, routine preservation of ARM‐identified nodes is not appropriate in such patients.

Thus, it is important to identify patients who will benefit from ARM. In addition to nodal distributions, we must acknowledge that metastasis largely occurs in conjunction with late clinical staging (III) and extensive axillary involvement (pN3), consistent with past reports of metastases in ARM‐identified nodes.^14^ Similarly, we found that shape, size, and texture of ARM‐identified nodes correlate with the risk of metastasis. Positive nodes are typically rounded, firm, and larger in size (>1 cm).

To better preserve uninvolved ARM‐identified nodes, we checked for metastasis before ALND using FNAC. This approach proved quite effective, achieving a sensitivity of 100% and a specificity of 86.4%, consistent with previous findings.^18^ Unfortunately, FNAC is hampered by occasional inadequate sampling, which in our patients accounted for 11.9% (10/84) of the specimens. These results are comparable with or lower than those obtained in other studies^18^, ^33^ and can be remedied by repeated samplings.

In summary, ICG is advantageous in ARM, enabling a higher identification rate than that achieved with MB with fewer complications in the prospective randomized study. FNAC is safe and efficient to assess the metastasis of ARM‐identified node in this setting and is recommended to target nodes for removal during ALND. The ARM with ICG may provide an approach suitable to preserve the nodes identified for patients with clinically positive nodes. However, one limitation of this study is unable to evaluate the effect of ARM in preventing lymphedema because all ARM‐identified nodes were removed during ALND. Therefore, additional prospective study is needed to evaluate whether ARM could help to curtail lymphedema effectively based on the feasibility and oncologic safety of ARM by ICG.

## CONFLICT OF INTEREST

The authors declared no conflict of interest.

## AUTHOR CONTRIBUTION

Jun‐Dong Wu directed and performed the clinical study. Zun Wang performed data analysis and wrote the manuscript. Huan‐Cheng Zeng, Li‐Fang He, Yong‐Qu Zhang, Guang‐Sheng Huang, and Wen‐He Huang performed patient enrollment, conducted the clinical study, and contributed to the clinical specimen and data collection. Fan Zhang conducted statistical analysis for the clinical data. Xiao‐Long Wei performed pathologic evaluation. Guo‐Jun Zhang designed and directed the study and finalized the manuscript.

## Supporting information

Supporting Tables

Figure S1
