# Supplementary material for: Comparison of indocyanine green and methylene blue use for axillary reverse mapping during axillary lymph node dissection
Source: MedComm (2020). 2020 Sep 17;1(2):211–8. doi: 10.1002/mco2.31 (PMC8491232; doi:10.1002/mco2.31)
Supplement: Supplementary file 1 — Supporting Tables [file MCO2-1-211-s001.docx]

**Supplementary tables:**

**Supplementary Table 1.** Clinicopathologic characteristics of patients by group (mean ± SD^a^)

|  | **ICG^b^ group**  **(n=78)** | **MB^c^ group**  **(n=80)** | **p value** |
| --- | --- | --- | --- |
| BMI^d^ | 21.27 ± 2.62 | 20.74 ± 2.42 | .19 |
| Age | 54.76 ± 10.94 | 53.01 ± 8.78 | .27 |
| Clinical T classification |  |  | .87 |
| T1 | 12 (15.4) | 15 (18.8) |  |
| T2 | 55 (70.5) | 52 (65.0) |  |
| T3 | 6 (7.7) | 6 (7.5) |  |
| T4 | 5 (6.4) | 7 (8.8) |  |
| Clinical N classification |  |  | .12 |
| cN0 | 28 (35.9) | 17 (21.3) |  |
| cN1 | 35 (44.9) | 49 (61.3) |  |
| cN2 | 10 (12.8) | 7 (8.8) |  |
| cN3 | 5 (6.4) | 7 (8.8) |  |
| Histologic grade |  |  | .75 |
| Ⅰ | 5 (6.4) | 3 (3.8) |  |
| Ⅱ | 25 (32.1) | 31 (38.8) |  |
| Ⅲ | 44 (56.4) | 43 (53.8) |  |
| Unknown | 4 (5.1) | 3 (3.8) |  |
| Histotype |  |  | .43 |
| Ductal | 68 (87.2) | 74 (92.5) |  |
| Lobular | 3 (3.8) | 3 (3.8) |  |
| Other | 7 (9.0) | 3 (3.8) |  |
| ER^e^ status |  |  | .39 |
| Negative | 27 (34.6) | 33 (41.3) |  |
| Positive | 51(65.4) | 47 (68.7) |  |
| PR^f^ status |  |  |  |
| Negative | 40 (51.3) | 50 (62.5) | .15 |
| Positive | 38 (48.7) | 30 (37.5) |  |
| HER-2^g^ status |  |  | .53 |
| Negative | 52 (66.7) | 57 (71.3) |  |
| Positive | 26 (33.3) | 23 (28.7) |  |
| Ki-67 index |  |  | .24 |
| ≤14 | 11 (14.1) | 17 (21.3) |  |
| >14 | 67 (85.9) | 63 (78.7) |  |
| Molecular subtype |  |  | .57 |
| Luminal A | 4 (5.1) | 7 (8.8) |  |
| Luminal B | 48 (61.5) | 41 (51.3) |  |
| HER-2^g+^/ER^e-^/PR^f-^ | 14 (17.9) | 16 (20.0) |  |
| Triple negative | 12 (15.4) | 16 (20.0) |  |
| Clinical stage |  |  | .93 |
| Ⅰ | 14 (17.9) | 15 (18.8) |  |
| Ⅱ | 49 (62.8) | 48 (60.0) |  |
| Ⅲ | 15 (19.2) | 17 (21.3) |  |
| NAC^h^ |  |  | .76 |
| Yes | 16 (20.5) | 18 (22.5) |  |
| No | 62 (79.5) | 62 (77.5) |  |

Data expressed as mean±SD or numerical value (%), ^a^ Standard Deviation, ^b^ Indocyanine Green, ^c^ Methylene Blue, ^d^ Body Mass Index, ^e^ Estrogen Receptor, ^f^ Progesterone Receptor, ^g^ Human Epidermal Growth Factor Receptor, ^h^ Neoadjuvant Chemotherapy

**Supplementary Table 2.** Impact of NAC^a^ on ARM^b^ nodal identification rates by group

|  | **ICG^c^ group** | | | **MB^d^ group** | | |
| --- | --- | --- | --- | --- | --- | --- |
|  | NAC  (n=16) | No NAC  (n=62) | p value | NAC  (n=18) | No NAC  (n = 62) | p value |
| Identified | 15 (93.8) | 53 (85.5) | .644 | 8 (44.4) | 34 (54.8) | .437 |
| Unidentified | 1 (6.2) | 9 (14.5) |  | 10 (55.6) | 28 (45.2) |  |

Data expressed as numerical value (%), ^a^ Neoadjuvant Chemotherapy, ^b^ Axillary Reverse Mapping, ^c^ Indocyanine Green, ^d^ Methylene Blue.

**Supplementary Table 3.** Effect of BMI^a^ on the Identification Rates of ARM^b^ Nodes in ICG^c^ and MB^d^ Groups

|  | **ICG^c^ group** | |  | **MB^d^ group** | |  |
| --- | --- | --- | --- | --- | --- | --- |
|  | Identification | Failed Identification | p value | Identification | Failed Identification | p value |
|  | (n = 68) (%) | (n = 10) (%) |  | (n = 42) (%) | (n = 38) (%) |  |
| BMI^a^ |  |  | .573 |  |  | .140 |
| <18.5 | 3(4.4) | 1(10.0) |  | 4(9.5) | 9(23.7) |  |
| 18.5-24.9 | 59(86.7) | 9(90.0) |  | 36(85.7) | 25(65.8) |  |
| 25-29.9 | 5(7.4) | 0(0) |  | 2(4.8) | 3(7.9) |  |
| >30 | 1(1.5) | 0(0) |  | 0(0) | 1(2.6) |  |

^a^ Body Mass Index(kg/m2), ^b^ Axillary Reverse Mapping, ^c^ Indocyanine Green, ^d^ Methylene Blue.

**Supplementary Table 4.** Concordance between FNAC^a^ and histologic findings in ARM^b^-identified nodes

|  | **FNAC^a^** | **Histopathology** |
| --- | --- | --- |
|  | **(n = 74)** | **(n = 74)** |
| Negative | 51 (68.9) | 59 (79.7) |
| Positive | 12 (16.2) | 15 (20.3) |
| Suspicious | 11 (14.9) |  |

Data expressed as numerical value (%), ^a^ fine needle aspiration cytology, ^b^ axillary reverse mapping.

**Supplementary Table 5.** Complications of ARM^a^ using ICG^b^ or MB^c^

| **Parameter, n (%)** | **ICG group (n = 78)** | **MB group**  **(n = 80)** | **p value** |
| --- | --- | --- | --- |
| Skin tattoo | 49 (62.8) | 73 (91.3) | .13 |
| Pain at injection site | 17 (21.8) | 57 (71.3) | < .001*** |
| Local skin reaction | 22 (28.2) | 53 (66.3) | .004** |
| Induration | 3 (3.8) | 46 (57.5) | < .001*** |

^a^ axillary reverse mapping, ^b^ indocyanine green, ^c^ methylene blue. *p < .05, **p < .01, ***p < .001
