# Supplementary material for: Comparison of indocyanine green and methylene blue use for axillary reverse mapping during axillary lymph node dissection
Source: MedComm (2020). 2020 Sep 17;1(2):211–8. doi: 10.1002/mco2.31 (PMC8491232; doi:10.1002/mco2.31)
Supplement: Supplementary file 2 — Figure S1 [file MCO2-1-211-s002.docx]

**Supplementary figure:**


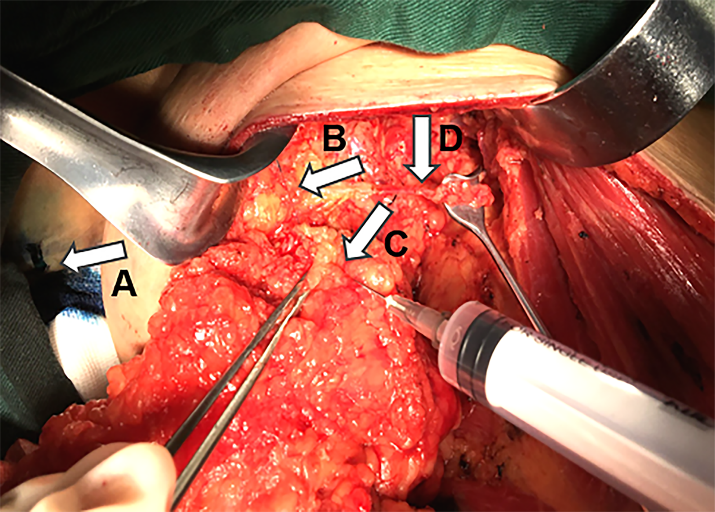


**Figure S1**. Intraoperative fine-needle aspiration cytology of axillary reverse mapping (ARM)-identified nodes: Arrow A, methylene blue injection site; Arrow B, ARM of lymphatic duct; Arrow C, ARM of lymph nodes; Arrow D, second intercostal brachial nerve.
